# Loss-of-function of p53 isoform Δ113p53 accelerates brain aging in zebrafish

**DOI:** 10.1038/s41419-021-03438-9

**Published:** 2021-02-04

**Authors:** Ting Zhao, Shengfan Ye, Zimu Tang, Liwei Guo, Zhipeng Ma, Yuxi Zhang, Chun Yang, Jinrong Peng, Jun Chen

**Affiliations:** 1 MOE Key Laboratory of Biosystems Homeostasis & Protection and Innovation Center for Cell Signaling Network, College of Life Sciences, Zhejiang University, 310058 Hangzhou, China; 2 College of Animal Sciences, Zhejiang University, 310058 Hangzhou, China; 3 Present Address: National Clinical Research Center for Child Health, The Children’s Hospital, Zhejiang University School of Medicine, 310052 Hangzhou, China

**Keywords:** Ageing, Cell proliferation

## Abstract

Reactive oxygen species (ROS) stress has been demonstrated as potentially critical for induction and maintenance of cellular senescence, and been considered as a contributing factor in aging and in various neurological disorders including Alzheimer’s disease (AD) and amyotrophic lateral sclerosis (ALS). In response to low-level ROS stress, the expression of Δ133p53, a human p53 isoform, is upregulated to promote cell survival and protect cells from senescence by enhancing the expression of antioxidant genes. In normal conditions, the basal expression of Δ133p53 prevents human fibroblasts, T lymphocytes, and astrocytes from replicative senescence. It has been also found that brain tissues from AD and ALS patients showed decreased Δ133p53 expression. However, it is uncharacterized if Δ133p53 plays a role in brain aging. Here, we report that zebrafish Δ113p53, an ortholog of human Δ133p53, mainly expressed in some of the radial glial cells along the telencephalon ventricular zone in a full-length p53-dependent manner. EDU-labeling and cell lineage tracing showed that *Δ113p53*-positive cells underwent cell proliferation to contribute to the neuron renewal process. Importantly, *Δ113p53*^*M/M*^ mutant telencephalon possessed less proliferation cells and more senescent cells compared to wild-type (WT) zebrafish telencephalon since 9-months old, which was associated with decreased antioxidant genes expression and increased level of ROS in the mutant telencephalon. More interestingly, unlike the mutant fish at 5-months old with cognition ability, *Δ113p53*^*M/M*^ zebrafish, but not WT zebrafish, lost their learning and memory ability at 19-months old. The results demonstrate that *Δ113p53* protects the brain from aging by its antioxidant function. Our finding provides evidence at the organism level to show that depletion of Δ113p53/Δ133p53 may result in long-term ROS stress, and finally lead to age-related diseases, such as AD and ALS in humans.

## Introduction

Reactive oxygen species (ROS), such as superoxide anion (O_2_•^–^), hydroxyl radical (OH•) and the nonradical species hydrogen peroxide (H_2_O_2_), are generated endogenously such as in the process of mitochondrial oxidative phosphorylation, or they may arise from interactions with exogenous sources such as xenobiotics, cytokines and bacterial invasion^1–3^. ROS is a double-edged sword for cell fate determination. ROS at moderate levels is essential for normal cellular signaling, whereas high levels and long-time exposure of ROS can oxidize cellular macromolecules such as DNA, lipids, and proteins, ultimately resulting in abnormal cell death and senescence^4^. A large amount of oxygen being consumed in the brain leads to excessive production of ROS. Most neuron cells have a large membrane being enriched in polyunsaturated fatty acids, which are highly susceptible to ROS^4,5^. Therefore, the brain becomes prone to oxidative stress that is proposed as a regulatory factor in aging and the progression of multiple neurodegenerative diseases including Alzheimer’s disease (AD) and amyotrophic lateral sclerosis (ALS)^5–8^.

The signaling pathway of the tumor repressor p53 plays a key role in response to oxidative stress^9–12^. Under low levels of ROS, p53 transcribes antioxidant genes to maintain redox homeostasis and promote cell survival, whereas, in response to high levels of oxidative stress, p53 triggers apoptotic activity by upregulating the expression of pro-oxidative genes and apoptotic genes^11,13–15^. Human p53 encodes at least 12 isoforms^16,17^. Δ133p53/Δ113p53 (its zebrafish ortholog) is an N-terminal truncated isoform and a p53 target gene transcribed from an alternative *p53* promoter in intron 4 (ref. ^18^). In response to DNA damage, Δ133p53/ Δ113p53 is upregulated to repress cell apoptosis by differentially modulating the expression of p53 target genes and to coordinate with p73 to promote DNA double-strand break repair by upregulating the transcription of repair genes^19–21^. In response to sub-toxic ROS stresses, Δ133p53 is also induced to promote cell survival and prevent senescence by coordinating with full-length p53 to transcribe antioxidant genes^22^. Our recent study revealed that zebrafish *Δ113p53* is induced by heart injury to promote heart regeneration by maintaining redox homeostasis^23^. The basal expression of Δ133p53 prevents normal human fibroblasts, T lymphocytes, and astrocytes from replicative senescence by repressing *p21* and *miR-34a* expression^24–26^. Interestingly, decreased Δ133p53 expression has been observed not only in replicative senescent cells but also in brain tissues from AD and ALS patients^26^.

Our previous studies have shown that *Δ113p53*^*M/M*^ mutant fish have normal development and growth though they are more sensitive to DNA damage stresses and have defects in heart regeneration^20,23^. Therefore, it is interesting to know in what kinds of zebrafish brain cells Δ113p53 expresses and if it plays a role in brain aging at the normal condition. Zebrafish has been widely applied to understand stem cell activity in the brain and the molecular processes required for regeneration of the central nervous system (CNS)^27^. Previous studies have revealed that the zebrafish brain does not have astrocytes, and has radial glia cells instead^28^. Zebrafish radial glia cells are regarded to be the adult neural stem cells (NSCs) throughout life and also serve some specialized roles of astrocytes in mammals^29–31^. Proliferation zones in the adult zebrafish brain are located in distinct regions along its entire anterior–posterior axis^32^. Zebrafish has the widespread adult neurogenesis ability along the brain axis which contributes to NSC diversity and brain regeneration^27,33^. In zebrafish telencephalon, different stem cell niches including Nestin-positive neuroepithelial-like progenitors, radial glial progenitors, and others, are distributed mainly along the ventral and dorsal of telencephalon ventricle, which constitutively give rise to different subtypes of neurons^32–36^. The proliferation cells along the ventral region of the ventricle migrate rapidly to its parenchymal tissue at a long distance, whereas the proliferation cells along the dorsal region of the ventricle move a small distance to the adjacent and subjacent telencephalic nuclei^32^. In addition, some cells can migrate into the olfactory bulb through the rostral migratory stream (RMS)^36,37^. The progenitor cell proliferation permits the zebrafish brain to undergo neurogenesis and replenish the lost cells after injury^33,38^.

In this study, we used the zebrafish model to investigate the function of Δ113p53 in brain aging. Here, we found that *Δ113p53* expressed only in a subgroup of the radial glia cells and RMS cells along the telencephalon ventricular zone (VZ). *Δ113p53*^*M/M*^ mutant zebrafish had an elevated level of ROS in its telencephalon and displayed accelerated brain aging phenotypes. Our results suggest that decreased Δ133p53 expression in astrocytes may result in long-term ROS stress, and finally leads to AD and ALS diseases in human.

## Results

### *Δ113p53* mainly expresses in radial glia cells along the ventricular zone in the zebrafish telencephalon

**Fig. 1:**
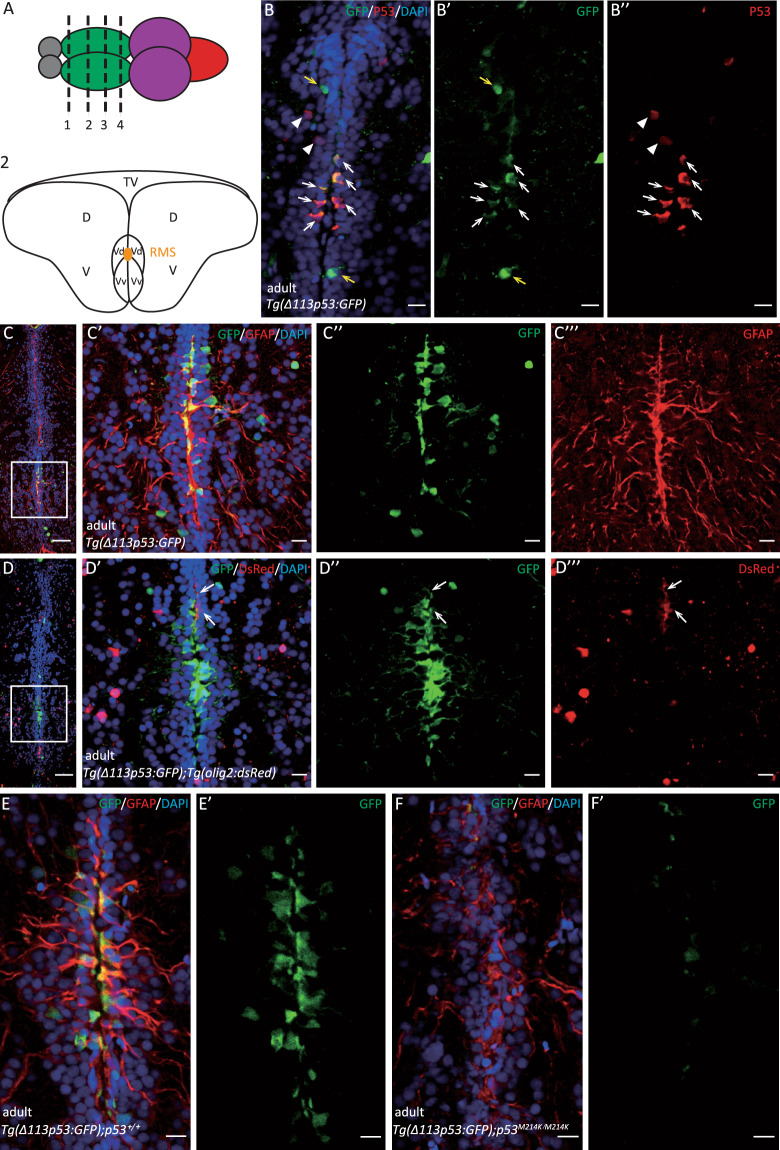
Most of *Δ113p53* expresses in the radial glial cells and a small part in RMS cells along the telencephalon ventricular zone. **A** Top panel: a **s**chematic diagram representing zebrafish brain structure. The dotted lines with different numbers represent different positions in the zebrafish telencephalon along the anterior to the posterior axis. Gray color: olfactory bulb; green color: telencephalon; purple color: midbrain; red color: hindbrain. Bottom panel: diagram representing the cross-section of telencephalon corresponding to the position as indicated by the dotted line 2 in the top panel. TV telencephalic ventricle, D dorsal telencephalic area, V ventral telencephalic area, Vd dorsal nucleus of V, Vv ventral nucleus of V, RMS rostral migratory stream. The photos in the following panels were representatives of the cross-sections corresponding to the region shown in the diagram. The representatives of other regions were presented in Supplementary Fig. S1. **B** Cryosections of *Tg(Δ113p53:GFP)* telencephalon were immunostained by anti-GFP (in green) (**B’**) and anti-p53 (in red) antibodies (**B”**). The nuclei were stained with DAPI (in blue). Arrowhead: p53^+^/GFP^−^ cells; yellow arrow: p53^-^/GFP^+^ cells; white arrow: p53^+^/GFP^+^ cells. Scale bar, 10 μm. **C** Cryosections of *Tg(Δ113p53:GFP)* telencephalon were immunostained by anti-GFP (in green) and anti-GFAP (in red) antibodies. The nuclei were stained with DAPI (in blue). The framed area in **C** was magnified in **C’** (merged), **C”** (GFP), and **C”’** (GFAP). Scale bar in **C**, 50 μm; Scale bar in **C’**, **C”**, **C”’**, 10 μm. **D** Cryosections of *Tg(Δ113p53:GFP;olig2:dsRed)* telencephalon were immunostained by anti-GFP (in green), and the red fluorescence was from the en vivo DsRed. The nuclei were stained with DAPI (in blue). The framed area in **D** was magnified in **D’** (merged), **D”** (GFP), and **D”’** (DsRed). White arrows: GFP^+^/DsRed^+^ cells. Scale bar in **D**, 50 μm; scale bar in **D’**, **D”**, **D”’**, 10 μm. **E**, **F** Cryosections of *Tg(Δ113p53:GFP);p53*^*+/+*^ telencephalon (**E**, **E’**) and *Tg(Δ113p53:GFP);p53*^*M214K/ M214K*^ telencephalon (**F**, **F’**) were immunostained by anti-GFP (in green) and anti-GFAP (in red) antibodies. The nuclei were stained with DAPI (in blue). Scale bar, 10 μm.

In order to investigate the expression of *Δ113p53* in the adult brain, *tg(Δ113p53:GFP)* transgenic zebrafish was applied, in which the expression of GFP faithfully mimics the transcription of endogenous *Δ113p53* (ref. ^39^). We found that the GFP signals were mainly distributed along VZ, especially enriched in the ventral nucleus of the ventral telencephalic area (Vv) and the dorsal nucleus of the ventral telencephalic area (Vd) in the anterior telencephalon, and extended to the anterior part of the parvocellular preoptic nucleus (PPa) and the postcommissural nucleus of the ventral telencephalic area (Vp) in the posterior of telencephalon (Fig. 1A, B and Supplementary Fig. S1). To confirm the expression of *Δ113p53*, we performed immunostaining with a zebrafish p53 antibody (recognizing both p53 and Δ113p53). The results showed that most of the GFP signals in the *tg(Δ113p53:GFP)* zebrafish telencephalon were co-localized with the endogenous p53 (Fig. 1B).

Zebrafish telencephalon plays an important role in neurogenesis and participates in cell proliferation, migration, and regeneration^32,36,37,40^. VZ is the proliferation zone in zebrafish telencephalon, in which there are abundant radial glia cells and RMS cells^41,42^. In this region, radial glia cell bodies are distributed along the VZ surface, and their long cytoplasmic processes extend to the pial surface, in which several marker genes (*gfap*, *blbp*, and *S100β*) express^30,35,43^. Most RMS cells are a group of migrating neuroblasts expressing *olig2* or *PSA-NCAM*^37,42,44^. To investigate what cell types the *Δ113p53-*positive cells are, we used a glial fibrillary acidic protein (Gfap) antibody to mark radial glia cells and a *tg(olig2:dsRed)* transgene (*dsRed* expression driven by an *olig2* promoter) or PSA-NCAM antibody to label RMS cells. Immunostaining from *tg(Δ113p53:GFP)* transgenic fish and *tg(Δ113p53:GFP;olig2:dsRed)* double-transgenic fish showed that most of *Δ113p53-*positive cells were co-stained with Gfap and small number of *Δ113p53-*positive cells co-localized with DsRed or PSA-NCAM (Fig. 1C, D and Supplementary Figs. S1B-E, S2). The results demonstrate that most of *Δ113p53-*positive cells are radial glia cells and some of them are RMS cells.

Our previous study showed that *Δ113p53* is a p53 target gene^39^. To determine if the expression of *tg(Δ113p53:GFP)* transgene was p53-dependent, we crossed the *tg(Δ113p53:GFP)* transgene into the *p53*^*M214K*^ mutant background, in which the transcriptional activity of mutant p53 is lost^45^. Unlike in WT brain, GFP was almost undetectable in the telencephalon of *p53*^*M214K*^ mutant brain (Fig. 1E, F). Taken together, the results demonstrate that *Δ113p53* expresses in some radial glia cells and RMS cells localized in the proliferation zone of zebrafish telencephalon.

### *Δ113p53*-positive radial glia cells undergo cell proliferation and contribute to the neuron renewal process

**Fig. 2:**
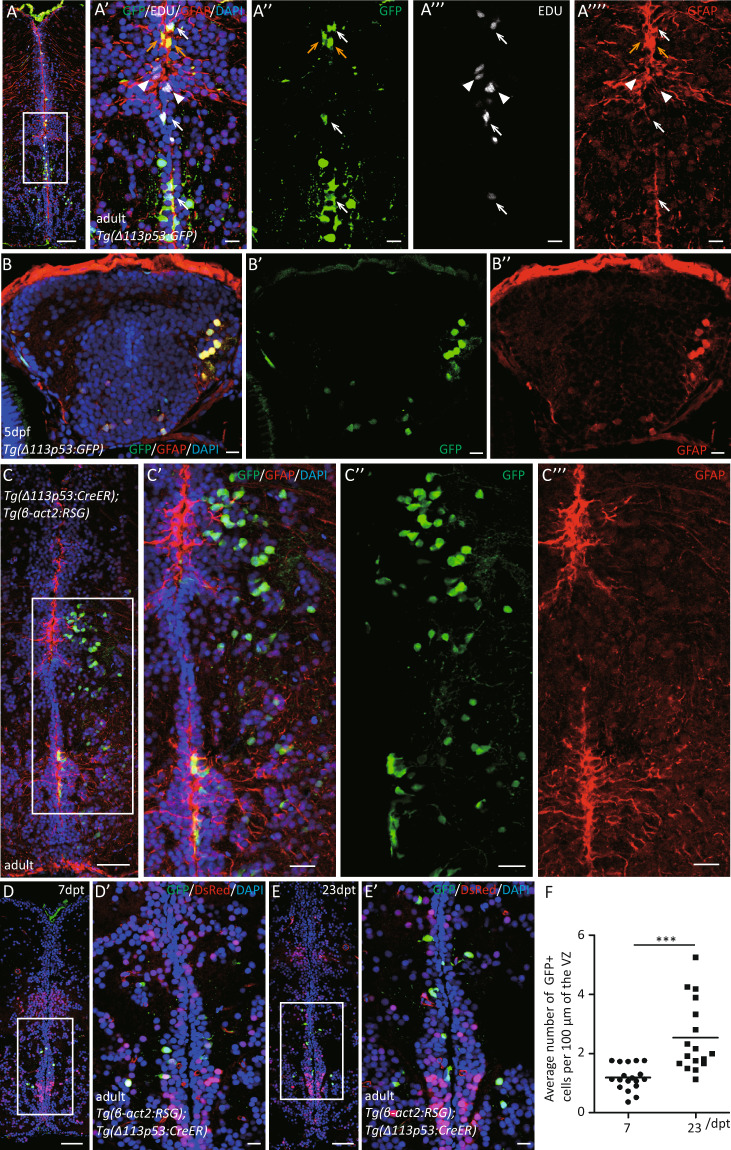
*Δ113p53*-positive radial glia cells undergo cell proliferation and contribute to the neuron renewal process. **A** Cryosections of EDU-labeled *Tg(Δ113p53:GFP)* telencephalon were immunostained by anti-GFP (in green), anti-GFAP (in red), labeled-EDU in white, and nuclei with DAPI (in blue). The framed area in **A** was magnified in **A’**, anti-GFP in **A”**, labeled-EDU in **A”’** and anti-GFAP in **A””**. White arrow: GFP^+^/GFAP^+^/EDU^+^ cells. Yellow arrows: GFP^+^/GFAP^+^ cells; white arrowhead: EDU^+^/GFAP^+^ cells. Scale bar in **A**, 50 μm; Scale bar in **A’**, **A”**, **A”’**, **A””**, 10 μm. **B** Cryosections of *Tg(Δ113p53:GFP)* larva brain at 5 dpf were immunostained by anti-GFP (in green) (**B’**) and anti-GFAP (in red) (**B”**) antibodies. Scale bar, 10 μm. **C**–**F** *Δ113p53*-positive cells in *Tg(Δ113p53:CreER;β-act2:RSG)* transgenic fish were genetically labeled at 5 dpf or at the adult stage by inducing Cre activity with 4-HT. The labeled larvae grew up to 6-months old and were subjected to immunostaining analysis (**C**), whereas the labeled adult fish were sampled at either 7 (**D**) or 23 dpt (**E**). Cryosections of labeled *Tg(Δ113p53:CreER;β-act2:RSG)* telencephalon were immunostained by anti-GFP (in green), anti-GFAP (in red) antibodies in **C**. In **D** and **E**, the red fluorescence was from the expression of en vivo DsRed. The nuclei were stained with DAPI (blue). The framed area in **C** was magnified in **C’** (merged)**, C”** (GFP), and **C”’** (GFAP). Framed areas in **D** and **E** were magnified in **D’** and **E’**, respectively. Scale bar in **C**, **D**, **E**, 50 μm; Scale bar in **C’**, **C”**, **C”’**, 20 μm; Scale bar in **E’**, **F’**, 10 μm. The average number of GFP^+^ radial glia cells per 100 μm along the ventricle zone of *Tg(Δ113p53:CreER; β-act2:RSG)* telencephalon at 7 and 23 dpt was represented in **F**. Each dot represents the average number of GFP^+^ radial glia cells per 100 μm of the ventricular zone (VZ) in one section. The middle region of each telencephalon (about three to six sections) was used for the counting and three telencephalons were sampled in each group. Statistical analysis was performed on relevant data using the Student’s two-tailed *t* test. ***P < 0.001.

In VZ, most radial glia cells alternate between a mitotic (Type II) and a quiescent state (Type I) for self-renew and damage repair. Type II radial glia cells can symmetrically divide into two radial glia cells, and also asymmetrically divide to self-renew and generate neuroblasts (Type III)^44^. To ask whether the *Δ113p53*-positive radial glia cells enter the mitotic state, we performed EDU (5-ethynyl-2′-deoxyuridine) labeling with *tg(Δ113p53:GFP)* transgenic fish. The results showed that some of the GFP-positive radial glia cells in VZ were labeled with EDU signals (Fig. 2A). Statistical analysis showed that there was no significant difference between the proportions of EDU-labeled GFP-positive radial glia cells (5.03%) and EDU-labeled total radial glia cells (6.92%) (Supplementary Fig. S3). The results suggest that *Δ113p53*-positive cells are a subgroup of radial glia cells, including proliferated and non-proliferated cells.

To explore whether *Δ113p53*-positive radial glia cells contribute to the neuronal renewal process, a cell lineage tracing assay was performed. The *tg(Δ113p53:CreER)* transgenic zebrafish generated with a 3.6-kb fragment of the *Δ113p53* promoter to drive CreER (tamoxifen-inducible Cre recombinase–estrogen receptor fusion protein) expression in our previous study was crossed with *tg(β-act2:RSG)* zebrafish to construct *tg(Δ113p53:CreER*; *β-act2:RSG)* double-transgenic fish^23^. The *tg(Δ113p53:GFP)* zebrafish larvae were used to investigate if *Δ113p53* expresses in radial glia cells at an early stage. We found that the GFP signals were observed in radial glia cells at 5 days post fertilization (dpf) (Fig. 2B). Therefore, we treated the *tg(Δ113p53:CreER; β-act2:RSG)* larvae with 4-hydroxytamoxifen (4-HT) at 5 dpf for 24 h and raised them to the adult stage. Interestingly, the GFP-positive cells were found not only along the VZ of 6-months-old zebrafish but also in the parenchyma of the telencephalon (Fig. 2C). Most of the GFP-positive cells in the parenchyma were not radial glia cells (Fig. 2C), suggesting that proliferated *Δ113p53*-positive radial glia cells migrated to other areas and differentiated into other cell types. To confirm *Δ113p53*-positive radial glia cells contribute to neurogenesis, we performed a similar assay in adult fish. The 6-months-old *tg(Δ113p53:CreER; β-act2:RSG)* fish were treated with 4-HT twice in 4 days (once at 1st and 4th day). Co-immunostaining showed that the number of GFP-positive cells along the VZ was significantly higher at 23 days post treatment (dpt) than that at 7 dpt (Fig. 2D–F). Taken together, these results demonstrate that partial *Δ113p53*-positive radial glia cells undergo cell proliferation and participate neuronal renewal process.

### Depletion of *Δ113p53* results in the elevated level of intracellular H_2_O_2_ in the zebrafish telencephalon

**Fig. 3:**
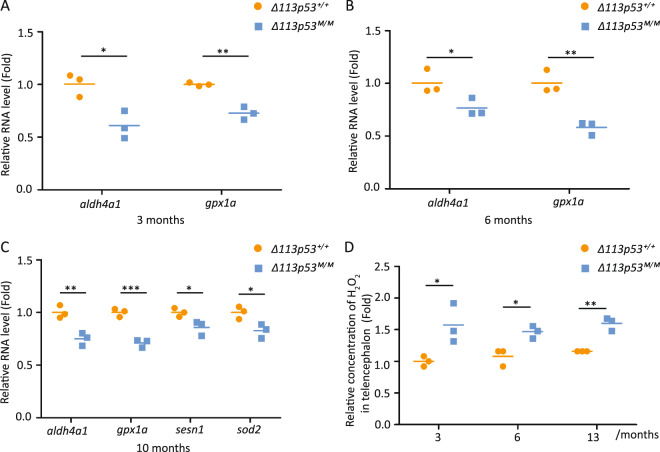
Depletion of *Δ113p53* results in an elevated level of intracellular H_2_O_2_ in zebrafish telencephalons. **A**–**C** Relative mRNA expression of *aldh4a1, gpx1a, sesn1, sod2* at 3- (**A**), 6- (**B**), and 10-months old (**C**) in the *Δ113p53*^*+/+*^ or *Δ113p53*^*M/M*^ telencephalons. The total RNA was extracted from a pool of at least six telencephalons in each group. The average gene expression was normalized against *β-actin* and expressed as fold change. **D** The relative concentration of H_2_O_2_ in the *Δ113p53*^*+/+*^ and *Δ113p53*^*M/M*^ telencephalons at 3-, 6-, and 13-months old as indicated. Each group was measured based on a pool of at least four telencephalons. Statistical analysis was performed on relevant data using the Student’s two-tailed *t* test. *P < 0.05, **P < 0.01, ***P < 0.001.

Our previous studies have demonstrated that the expression of either human *Δ133p53* or zebrafish *Δ113p53* is upregulated by low levels of ROS signals in cell culture and zebrafish heart regeneration respectively. Both of Δ133p53 and Δ113p53 function to promote cell survival or proliferation by upregulating the expression of antioxidant genes^22,23^. Therefore, we investigated whether the expression of *Δ113p53* is related to maintaining redox homeostasis in the zebrafish brain. For this purpose, we analyzed the expression of six antioxidant genes (p53 target genes) in WT and *Δ113p53*^*M/M*^ mutant telencephalons at different ages. The *Δ113p53*^*M/M*^ mutant with relatively normal development was generated in our previous study by creating an 11-bp deletion in a p53 responsive element in the *Δ113p53* promoter located in the 4th intron of *p53*, which abolishes the expression of *Δ113p53* but does not influence the expression of full-length p53 (ref. ^20^). To evaluate the expression of *Δ113p53* in the *Δ113p53*^*M/M*^ mutant telencephalons, we performed quantitative reverse transcription PCR (qRT-PCR) assay. The result showed that the expression of *Δ113p53* was about 90 times lower in the mutant telencephalons than that in WT telencephalons (Supplementary Fig. S4). Among the six antioxidant genes (*gpx1a*, *aldh4a1*, *sesn1, sesn2, sod1*, and *sod2*), the expression of *gpx1a* and *aldh4a1* was significantly lower in *Δ113p53*^*M/M*^ telencephalons than that in WT at the ages of 3- and 6-months old, whereas the expression of rest of four genes (*sesn1, sesn2, sod1, sod2*) had no significant changes between WT and *Δ113p53*^*M/M*^ telencephalons (Fig. 3A, B; Supplementary Fig. S5A, B). As fish grew up to 10-months old, the expression of up to four genes (*gpx1a*, *aldh4a1*, *sesn1*, and *sod2*) was significantly lower in *Δ113p53*^*M/M*^ telencephalons than that in WT (Fig. 3C and Supplementary Fig. S5C).

To further explore if the decreased expression of antioxidant genes in *Δ113p53*^*M/M*^ telencephalons impaired redox homeostasis, we performed an assay on the levels of H_2_O_2_ in WT and *Δ113p53*^*M/M*^ telencephalons at the ages of 3-, 6-, and 13-months old. The results showed that the concentration of H_2_O_2_ was significantly higher in the *Δ113p53*^*M/M*^ telencephalons at each of the three time points (3-, 6-, and 13-months old) than that in WT respectively (Fig. 3D). The data demonstrate that *Δ113p53* maintains redox homeostasis in the telencephalon by promoting the expression of antioxidant genes.

### *Δ113p53*^*M/M*^ zebrafish telencephalons contain more senescent cells

**Fig. 4:**
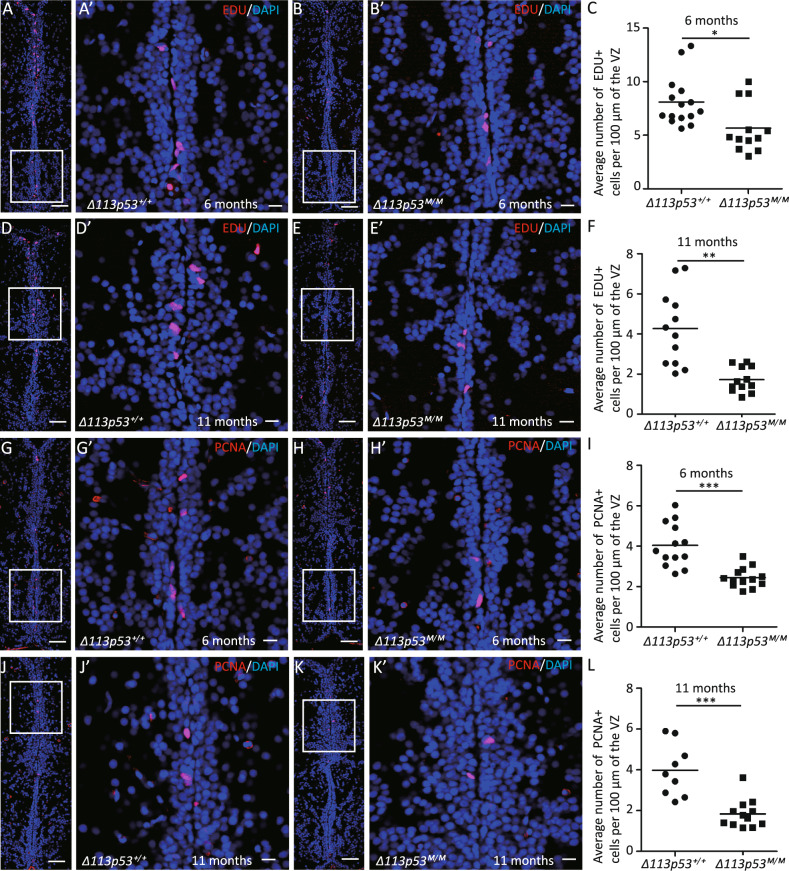
*Δ113p53* promotes cell proliferation in the ventricular zone of the telencephalon. **A**–**F** The *Δ113p53*^*+/+*^ (**A**, **D**) and *Δ113p53*^*M/M*^ (**B**, **E**) zebrafish telencephalons were labeled with EDU in red at 6- and 11-months old, as indicated. **G**–**L** Cryosections of *Δ113p53*^*+/+*^ (**G**, **J**) and *Δ113p53*^*M/M*^ (**H**, **K**) zebrafish telencephalons were immunostained by anti-PCNA (in red) at 6- and 11-months old, as indicated. The nuclei were stained with DAPI (in blue). An average number of EDU^+^ cells or PCNA^+^ cells per 100 μm of the ventricular zone (VZ) in one section from the middle region of each telencephalon was presented in **C** (6-months old) and **F** (11-months old) or **I** (6-months old) and **L** (11-months old), respectively. Framed areas in **A**, **B**, **D**, **E**, **G**, **H**, **J**, **K** were magnified in **A’**, **B’**, **D’**, **E’**, **G’**, **H’**, **J’**, **K’**, respectively. Scale bar in **A**, **B**, **D**, **E**, **G**, **H**, **J**, **K**, 50 μm; scale bar in **A’**, **B’**, **D’**, **E’**, **G’**, **H’**, **J’**, **K’**, 10 μm. Each dot represents the average number of EDU^+^ cells or PCNA^+^ cells per 100 μm of VZ in one section. About three to five sections were chosen from the middle region of each telencephalon and at least three telencephalons were sampled in each group. Statistical analysis was performed on relevant data using the Student’s two-tailed *t* test. *P < 0.05, **P < 0.01, ***P < 0.001.

To address if elevated ROS level in the *Δ113p53*^*M/M*^ telencephalons had an influence on cell cycle and cellular senescence, we performed EDU-labeling and immunostaining of PCNA (an S-phase marker) in WT and *Δ113p53*^*M/M*^. The EDU-labeling assay showed that the proportion of EDU-labeled cells was significantly lower in the VZ of *Δ113p53*^*M/M*^ telencephalons than that in WT at both of 6- and 11-months old (Fig. 4A–F). The results demonstrate that lack of *Δ113p53* leads to decreased proliferation of cells in the VZ of the telencephalon, which was confirmed in the PCNA-immunostaining experiment (Fig. 4G–L).

**Fig. 5:**
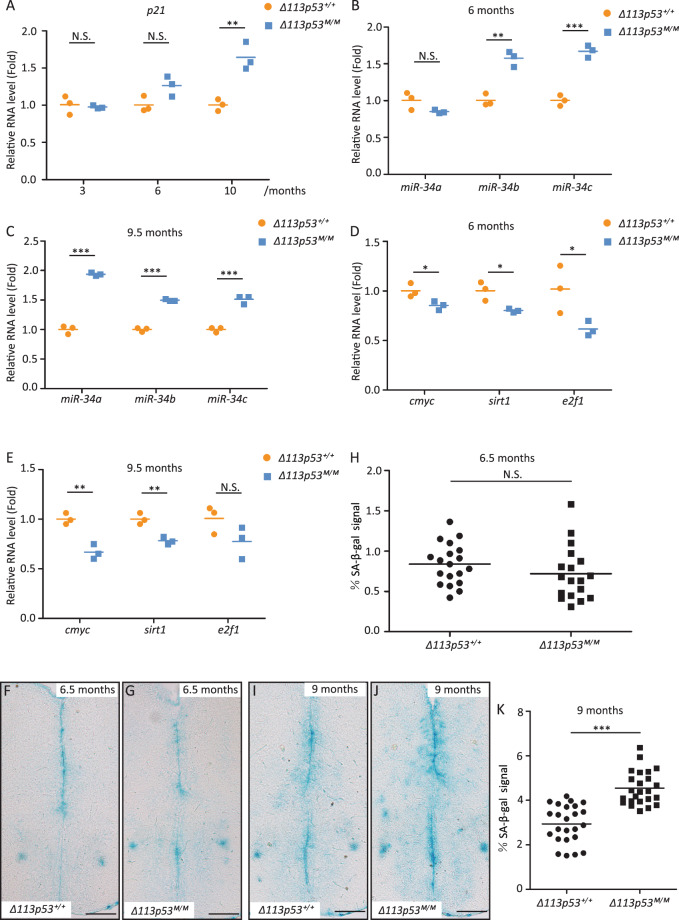
*Δ113p53*^*M/M*^ zebrafish telencephalon has increased levels of cell senescence markers. **A**–**E** Relative expression of *p21*(**A**), *cmyc*, *sirt1*, *e2f1* mRNAs (**D**, **E**), and *miR-34a*, *miR-34b*, *miR-34c* miRNAs (**B**, **C**) at different ages in the *Δ113p53*^*+/+*^ and *Δ113p53*^*M/M*^ telencephalons, as indicated. The total RNA was extracted from a pool of at least six telencephalons in each group. The average gene expression of mRNAs and miRNAs was normalized against *β-actin* and *U6*, respectively. **F**–**K** Senescence-associated β-galactosidase (SA-β-gal) staining in the telencephalons of *Δ113p53*^*+/+*^ (**F**, **I**) and *Δ113p53*^*M/M*^ zebrafish (**G**, **J**) at 6.5- and 9-months old as indicated. Scale bar, 100 μm. The average SA-β-gal signal was quantified with Photoshop and presented as the percentage of pixels per unit area of the section measured in the telencephalon (**H**, **K**). Each dot represents the average SA-β-gal signal in each section. About three to five sections were taken from the middle region of each telencephalon, and at least four telencephalons were sampled in each group. Statistical analysis was performed on relevant data using the Student’s two-tailed *t* test. N.S., *P* > 0.05, *P < 0.05, **P < 0.01, ***P < 0.001.

A study from human cell lines showed that the Δ133p53 knockdown induced senescence was accompanied by the upregulation of *p21* and *miR-34a*^24^. Therefore, we determined the expression of *p21, miR-34* family members (*miR-34a*, *miR-34b*, *miR-34c*), and three target genes of *miR-34* (*sirt1*, *e2f1,* and *cmyc*) in WT and *Δ113p53*^*M/M*^ telencephalons. qRT-PCR showed that the expression of *p21* and *miR-34a* remained similar level in *Δ113p53*^*M/M*^ telencephalons as that in WT before 6-months old, and increased significantly in *Δ113p53*^*M/M*^ telencephalons compared to that in WT telencephalons at 10- or 9.5-months old, whereas the expression of *miR-34b* and *miR-34c* was significantly higher in *Δ113p53*^*M/M*^ telencephalons than that in WT from 6-months old (Fig. 5A–C). In contrast, the expression of almost all of three *miR-34* family-target genes (*sirt1*, *e2f1, cmyc*) was significantly lower in *Δ113p53*^*M/M*^ telencephalons than that in WT from 6-months old, except for the expression of *e2f1* at 9.5-months old (Fig. 5D, E).

Next, we performed a cell senescence analysis using senescence-associated β-galactosidase (SA-β-gal) staining. Although the average staining signal intensity in each section of the around middle region of telencephalon was not significantly different between WT and *Δ113p53*^*M/M*^ at 3- and 6.5-months old (Fig. 5F–H and Supplementary Fig. S6A–C), as fish grew older, the average staining signal intensity in each section was significantly higher in *Δ113p53*^*M/M*^ than that in WT at 9- and 22-months old (Fig. 5I–K and Supplementary Fig. S6D–F).

Taken together, the loss-of-function of *Δ113p53* decreased cell proliferation capacity, eventually resulting in cellular senescence. Such reduced neurogenesis is one of the physiological changes in the aging brain. The results from H_2_O_2_ assays and SA-β-gal staining also suggest that the functions of *Δ113p53* influence the whole region of VZ, rather than only the cells it expresses.

### Depletion of *Δ113p53* has no effects on DNA damage response and apoptotic activity in zebrafish telencephalons

**Fig. 6:**
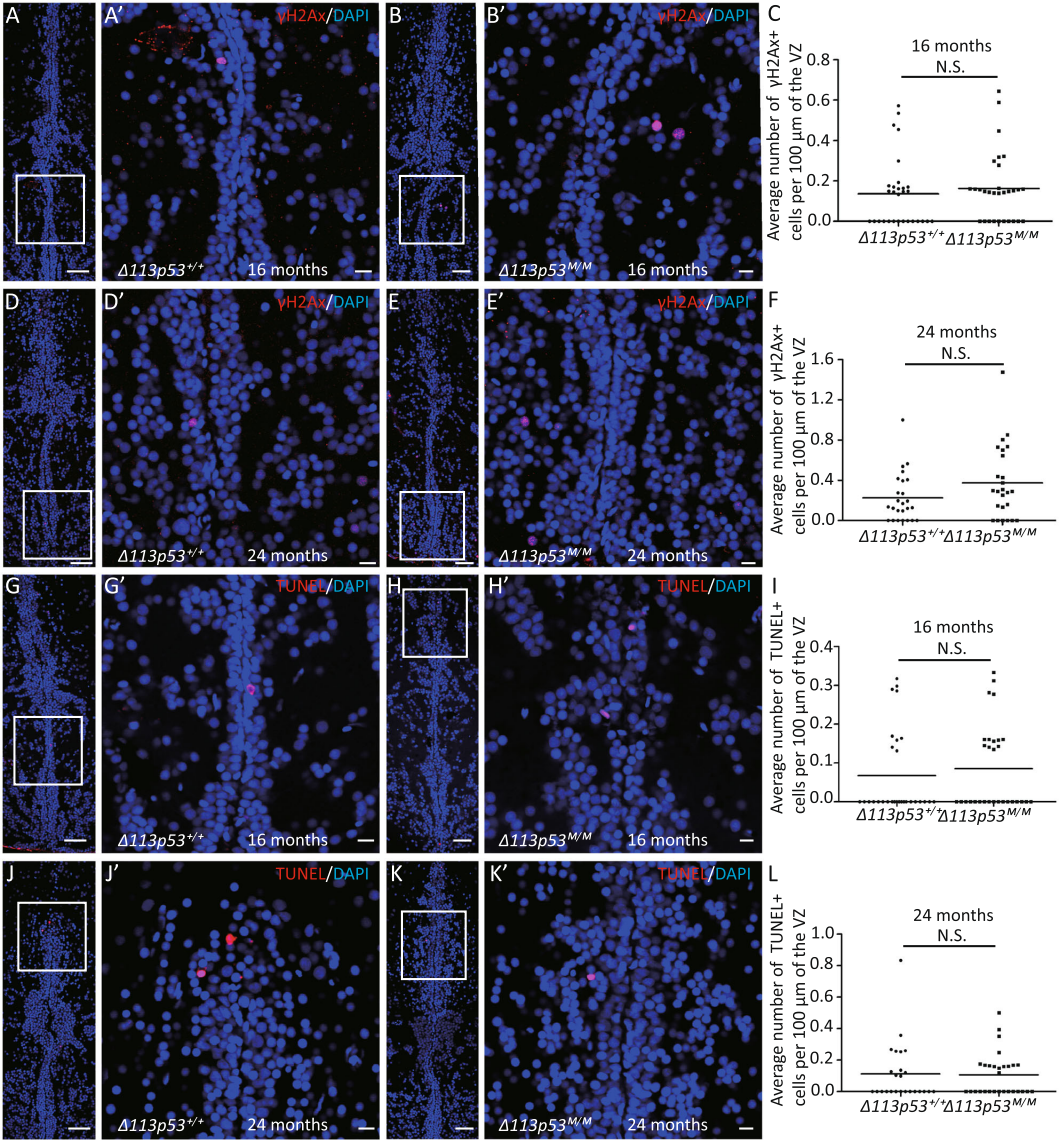
Depletion of Δ113p53 has little effect on DNA damage response and apoptotic activity in zebrafish telencephalons. **A**–**F** Cryosections of *Δ113p53*^*+/+*^ (**A**, **D**) and *Δ113p53*^*M/M*^ telencephalons (**B**, **E**) at 16- or 24-months old were stained with an anti-γ-H2AX (in red) antibody. **G**–**L** TUNEL assay (in red) was performed on cryosections of *Δ113p53*^*+/+*^ (**G**, **J**) and *Δ113p53*^*M/M*^ zebrafish telencephalons (**H**, **K**) at 16- and 24-months old, as indicated. The nuclei were stained with DAPI (in blue). An average number of γ-H2AX ^+^ cells or apoptotic cells per 100 μm of the ventricular zone (VZ) in one section from the middle region of each telencephalon was presented in **C** (16-months old) and **F** (24-months old) or **I** (16-months old) and **L** (24-months old), respectively. Framed areas in **A**, **B**, **D**, **E**, **G**, **H**, **J**, **K** were magnified in **A’**, **B’**, **D’**, **E’**, **G’**, **H’**, **J’**, **K’**, respectively. Scale bar in **A**, **B**, **D**, **E**, **G**, **H**, **J**, **K**, 50 μm; Scale bar in **A’**, **B’**, **D’**, **E’**, **G’**, **H’**, **J’**, **K’**, 10 μm. Each dot represents the average number of γ-H2AX ^+^ cells or apoptotic cells per 100 μm of VZ in one section. About four to five sections were chosen from the middle region of each telencephalon, and at least five telencephalons were sampled in each group. Statistical analysis was performed on relevant data using the Student’s two-tailed *t* test. N.S., *P* > 0.05.

Our previous studies have shown that the induction of Δ113p53 functions to antagonize p53-mediated apoptosis and promote DNA damage repair^20,39^. Therefore, we performed a TUNEL assay and immunostaining for γ-H2AX (an early marker of the DNA damage response) to analyze apoptotic cells and the DNA damage response in telencephalons, respectively. Only a few γ-H2AX-positive cells and apoptotic cells were observed in both WT and *Δ113p53*^*M/M*^ mutant telencephalons at 16- and 24-months old (Fig. 6A–L). There were no significant differences in the proportion of γ-H2AX-positive cells between WT and *Δ113p53*^*M/M*^ telencephalons, though the proportion of γ-H2AX-positive cells was slightly higher in the *Δ113p53*^*M/M*^ telencephalons at 24-months old than that in WT (Fig. 6A–F). The TUNEL assay also showed that both WT and *Δ113p53*^*M/M*^ mutant telencephalons had a similar level of apoptotic activity at either 16- or 24-months old (Fig. 6G–L). These results reveal that the absence of *Δ113p53* does not influence the DNA damage response and apoptotic activity in zebrafish telencephalons.

### Loss-of-function of *Δ113p53* impairs learning and memory capacity with aging

**Fig. 7:**
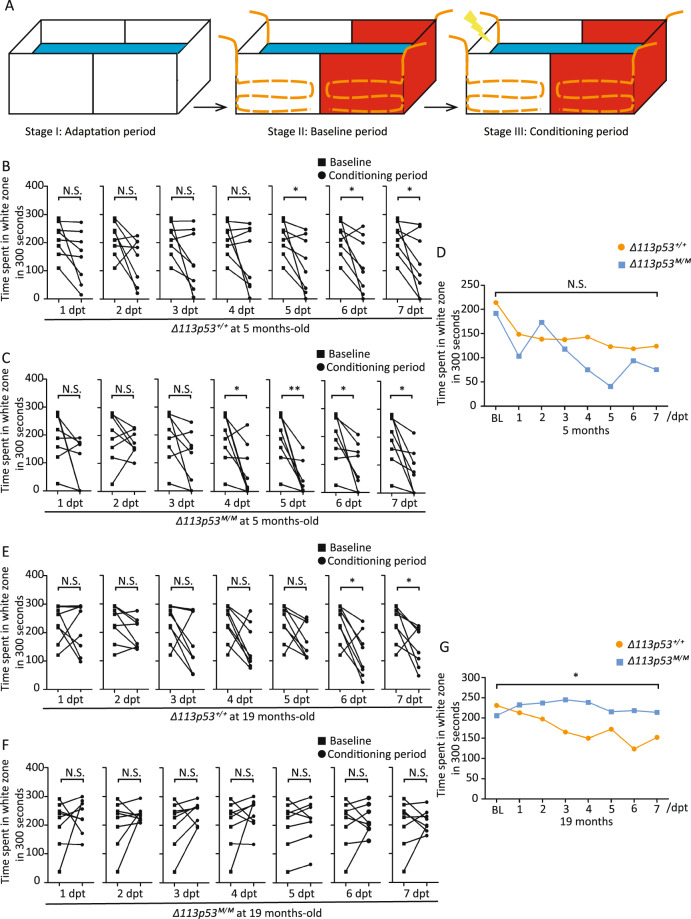
Loss-of-function of *Δ113p53* impairs learning and memory capacity with aging. **A** The schematic of the CPA paradigm. Stage I: Adaptation period in the tank covered with white color (5 min/day for 3 days); Stage II: Baseline period in the test tank covered by one side with red color and the other with white color (5 min/day for 6 days without electric shock); Stage III: Conditioning period in the test tank (5 min/day for 7 days after electric shock from white color). In stage II, zebrafish behavior was recorded for the Baseline period. In stage III, a mild electric shock for one min (twice/day in 1-h interval) was delivered each time the fish entered the white zone. Zebrafish behavior was recorded 1 day after the treatment for the conditioning period. After recording, the fish was treated again and the assay was repeated for 7 days. **B–G** Statistical analysis of CPA assays on *Δ113p53*^*+/+*^ and *Δ113p53*^*M/M*^ zebrafish at 5- or 19-months old. The paired *t* test was applied for the comparison of the time spent in the white color out of 300 s (=5 min) recorded between baseline and conditional periods within the same genotype either *Δ113p53*^*+/+*^ (**B**, **E**) or *Δ113p53*^*M/M*^ (**C**, **F**). Each dot represents the time for individual fish spending in the white zone. Two-way ANOVA analysis was performed to analyze the differences of the average time spent in the white color at each time point between two genotypes of *Δ113p53*^*+/+*^ and *Δ113p53*^*M/M*^ at 5- (**D**) or 19- (**G**) months old. BL Baseline period. In each group, eight zebrafish were used. Within-group comparison: paired *t* test. Between-group comparison: two-way ANOVA test. The *P* values were represented by N.S. and asterisks. N.S., *P* > 0.05, *P < 0.05, **P < 0.01.

Finally, we addressed if reduced neurogenesis in *Δ113p53*^*M/M*^ telencephalon caused effects on brain function. One of the age-associated alterations of brain function is cognitive decline, which includes deficits in learning and memory, vocabulary, conceptual reasoning, and processing speed^6^. Several sophisticated learning and memory paradigms have been developed for the zebrafish^46,47^. Here, we applied a negative reinforcement, namely, the conditioned place avoidance (CPA) paradigm, to determine the learning and memory capacity in young (<1-year old) and mid-aged (<2-year old) fish of WT and *Δ113p53*^*M/M*^. CPA is designed to evaluate the cognitive ability when zebrafish is adapted to a new experimental environment^46^. Briefly, the task design for CPA is as follow: the test tank was covered by two colors, one side with red color and the other with white color; following the adaptation period (3 days in the tank covered white color for 5 min/day), the fish was introduced to the test tank for 5 min/day in 6 days and zebrafish behavior was recorded for the baseline period; during the conditioning period, a mild electric shock for one min (twice/day in 1-h interval) was delivered each time the fish entered the white zone; the time fish spent in each half of the tank was recorded at 1 day after the treatment; after recording, the fish was treated again and the assay was repeated for 7 days (Fig. 7A).

No significant difference of the baseline period between WT (∼220 s/300 s in white color) and *Δ113p53*^*M/M*^ fish (∼200 s/300 s in white color) at either young (5-months old) or mid-aged (19-months old) stage was observed (Fig. 7D, G). Expectedly, both young (5-months old) WT and *Δ113p53*^*M/M*^ fish showed very fast development of avoidance of white color (a harmful stimulus), relative to the baseline. Compared to the baseline period, the time spent in the white zone was significantly reduced by the mild electric shock in WT fish at 5, 6, 7 dpt, and in *Δ113p53*^*M/M*^ group at 4, 5, 6, and 7 dpt, respectively (Fig. 7B, C), suggesting that it took 5 and 4 days for young WT and *Δ113p53*^*M/M*^ fish respectively to learn to avoid the white color (the average time for WT to spend in white color decreased significantly from 213 s/300 s down to 123 s/300 s at 5 dpt; the average time for *Δ113p53*^*M/M*^ fish to spend in white color decreased significantly from 191 s/300 s down to 75 s/300 s at 4 dpt) (Fig. 7B–D). However, there were no significant differences in the conditioning period between WT and *Δ113p53*^*M/M*^ fish (*P* > 0.05, two-way analysis of variance, ANOVA) (Fig. 7D). Interestingly, only mid-aged WT fish (the average time spent in white color decreased significantly from 230 s/300 s down to 123 s/300 s), but not mid-aged *Δ113p53*^*M/M*^ fish (the average time spent in white color was not decreased by the treatment of electric shock), developed ability to avoid white color after 6 dpt (Fig. 7E, F). ANOVA analysis showed that there was a significant difference between WT and *Δ113p53*^*M/M*^ fish in the conditioning period (Fig. 7G). The results demonstrate that loss-of-function of *Δ113p53* impairs cognitive capacity with aging.

## Discussion

The brain plays a central role in physiology and metabolism and may also be at the center of aging^6^. To accomplish its task, the brain consumes more energy than any other tissue in proportion to its size^5^. This high level of energy consumption can increase the production of ROS. Oxidative stress has been proposed to trigger neurodegenerative diseases and accelerate aging^3,5^. Our previous studies have revealed that human Δ133p53 is highly induced in response to low levels of ROS and functions to promote cell survival by promoting the expression of antioxidant genes, whereas zebrafish *Δ113p53* is induced by ROS signal during heart regeneration and promotes cardiomyocyte proliferation by maintaining redox homeostasis^22,23^. It has also been reported that human Δ133p53 expresses in astrocytes and prevents astrocytes from cell death and senescence. The expression of Δ133p53 decreases not only in cultured senescent astrocytes but also in brain tissues from AD and ALS patients^26^.

In this report, we used a model animal, zebrafish, to investigate the function of Δ113p53 in zebrafish brain aging. Based on a *Δ113p53* transgenic reporter fish and immunostaining, we found that, unlike the expression of Δ133p53 in mammal astrocytes, most of *Δ113p53* expressed in some of radial glia cells and a small part in RMS cells along the telencephalon VZ (Fig. 1C, D and Supplementary Figs. S1, S2). The expression of *Δ113p53* was almost not observed in *p53*^*M214K*^ mutant telencephalons (Fig. 1E, F), which is consistent with *Δ113p53* being a p53 target gene. Zebrafish radial glia cells have been demonstrated to be NSCs and have similar functions as astrocytes in mammals^28^. Therefore, the result suggests that zebrafish Δ113p53 may play a similar role as Δ133p53 in the human brain. EDU-labeling and cell lineage tracing experiments showed that *Δ113p53*-positive cells underwent cell proliferation and their progenies migrated to parenchymal tissues and differentiated into other cell types (Fig. 2A–F). Next, we explored the function of Δ113p53 in brain aging with *Δ113p53*^*M/M*^ mutants. RT-PCR showed that the expression of antioxidant genes decreased significantly in *Δ113p53*^*M/M*^ telencephalons, compared to that in WT telencephalons, which was associated with an increased level of H_2_O_2_ in the mutant telencephalons from 3-months old (Fig. 3A–D). Cell proliferation and senescent assays showed that the proportions of EDU-labeled and PCNA-positive cells were significantly lower in *Δ113p53*^*M/M*^ mutant telencephalons than those in WT telencephalons from 6-months old (Fig. 4A–L), whereas SA-β-gal activity significantly increased in whole VZ regions of *Δ113p53*^*M/M*^ mutant telencephalons, compared that in WT telencephalons from 9-months old (Fig. 5F–K). However, loss-of-function of Δ113p53 did not have effects on the DNA damage response and apoptotic activity in telencephalons (Fig. 6A–L).

The zebrafish telencephalon has been proved to control behaviorally driven social orienting, which is proposed to be homologous to mammalian subcortical structures that can regulate memory, emotion, and social behavior^48,49^. Therefore, we performed a CPA assay to investigate if loss-of-function of *Δ113p53* had effects on zebrafish behavior. The CPA assay revealed that the learning and memory ability significantly declined in mid-aged *Δ113p53*^*M/M*^ zebrafish, compared to that in mid-aged WT zebrafish (at 19-months old) (Fig. 7E–G), though young mutant fish had similar cognition capacity as WT fish did (at 5-months old) (Fig. 7B–D). The data demonstrate that Δ113p53 expresses in NSC-like cells to prevent brain aging by maintaining redox homeostasis.

Sirtuin1(Sirt1) is a nicotinamide adenine dinucleotide (NAD^+^)-dependent deacylase and plays protective roles in several neurodegenerative diseases^50^. Interestingly, we found that the expression of *sirt1* was downregulated in the *Δ113p53*^*M/M*^ telencephalon, which might be resulted from an elevated level of *miR-34* (Fig. 5B–E). A previous study showed that Δ133p53 represses the p53-dependent expression of *miR-34a*^24^. The *sirt1* mRNA is the target of *miR-34a*^51,52^. Therefore, in addition to increased ROS level, the decreased expression of *sirt1* may also contribute to brain aging in *Δ113p53*^*M/M*^ zebrafish.

Although a number of studies have demonstrated that Δ133p53/Δ113p53 plays roles in cell proliferation, apoptosis, senescence, DNA damage repair, and so on, Δ113p53 loss-of-function mutant zebrafish develop normal and are fertile without visible phenotypes^20,22,24,39^. Therefore, what physiological roles Δ133p53/Δ113p53 plays at the organism level are still elusive. In this report, we demonstrate that *Δ113p53* expresses only in the subgroup of radial glia cells and RMS cells along the VZ region. However, it functions to maintain redox homeostasis in the whole region of VZ. Depletion of Δ113p53 results in the elevated level of ROS in telencephalons. This long-term low level of ROS stress leads to cell senescence in the whole VZ region, and eventually causes loss-of-cognition ability at 19-months old. The age of 19-months old in zebrafish is around middle age. This finding with *Δ113p53*^*M/M*^ zebrafish mutant may provide the most possible evidence to demonstrate that long-term low level of ROS stress is the cause for loss of function of Δ133p53 in human astrocytes to develop age-related diseases, such as AD and ALS.

## Materials and methods

### Zebrafish strains

Zebrafish were raised and maintained in the standard units at Zhejiang University as described previously^20^. The *Δ113p53*^*M/M*^ mutant zebrafish^20^, *Tg(Δ113p53:GFP)*^39^ and *Tg(Δ113p53:CreER)*^23^ transgenic lines were generated in our previous studies. The *p53*^*M214K*^ mutant zebrafish^45^ and *Tg(β-act2:RSG)*^53^ transgenic lines were generated by different labs as previously reported. *Tg(olig2:dsRed)* was purchased from China Zebrafish Resource Center.

### Adult zebrafish telencephalon acquisition and cryosection

The zebrafish were anesthetized in 0.2‰ Tricaine and sacrificed in ice water. The whole zebrafish body was fixed in 4% PFA at 4 °C overnight. Then the telencephalon was isolated and cryosectioned (14 μm for immunostaining and 20 μm for SA-β-gal staining), as described previously.

### EDU incorporation assay

For the EDU incorporation assay, 3 μL of 0.5 M EDU (Invitrogen, A10044) was injected once daily into the abdominal cavity of each animal for 3 days. The telencephalons were then fixed for cryosection. EDU staining was performed using Azide Alexa Fluor 647 (Invitrogen, A10277).

### Cell lineage tracing experiment

For cell lineage tracing in the adult stage, the *tg(Δ113p53:CreER; β-act2:RSG)* zebrafish at 6-months old were bathed in the 3 μM 4-HT (Sigma, H7904) for 24 h under darkness. The treatment was repeated again with 2 days interval. The treated zebrafish was sampled at 7 and 23 dpt.

For cell lineage tracing in the larva stage, *tg(Δ113p53:CreER; β-act2:RSG)* larvae at 5 dpf were bathed in the 5 μM 4-HT for 24 h under darkness and grew up to 6-months old, subjected to immunostaining analysis.

### H_2_O_2_ detection

About four to six adult zebrafish telencephalons were homogenized. The H_2_O_2_ concentration assay was detected by using a ferrous ion oxidation-based Hydrogen Peroxide Assay Kit (Beyotime, S0038) according to the manufacturer’s introduction.

### SA-β-gal staining

The zebrafish were fixed in 4% PFA for 12 h at 4 °C. About three to six telencephalons in each group were isolated and washed for three times in PBS to subject to the SA-β-gal staining with the SA-β-gal staining kit (Beyotime, C0602) according to the manufacturer’s instructions. Stained telencephalons were cryosectioned. Images were taken under an Olympus BX53 microscope with a camera of Qimaging MicroPublisher 5.0 RTV. The average intensity of SA-β-gal signals in the VZ of each section within the middle region of telencephalon (about three to five additive sections/telencephalon) was calculated from total intensity being divided by the area of whole tissue in the section.

### Quantitative real-time reverse transcriptional PCR (qRT-PCR)

Zebrafish telencephalons were isolated immediately after anesthetization at different ages (3, 6, 10 months). The total RNA was extracted from six to eight telencephalons in each group using Trizol reagent (Invitrogen, 15596026). Isolated RNA was treated with DNaseI (NEB, M0303S) prior to reverse transcription and purified through lithium chloride.

For the analysis of mRNAs, the first-strand cDNA was synthesized using M-MLV Reverse Transcriptase (Invitrogen, C28025021). The quantitative PCR was performed with AceQ qPCR SYBR Green (Vazyme, Q111-02) using CFX96^TM^ Real-Time System (Bio-Rad) according to the manufacturer’s instructions. The total RNA levels were normalized to the level of *β-actin*. Statistics were obtained from three repeats. The primer sequences used in this study are listed in Supplementary Table S1.

For the analysis of miRNAs, the reverse transcription reaction was performed with miRcute Plus miRNA First-Strand cDNA Synthesis Kit (TIANGEN, KR211). The quantitative PCR was performed with miRcute Plus miRNA qPCR Detection Kit (TIANGEN, FP411) using CFX96^TM^ Real-Time System (Bio-Rad). The total RNA levels were normalized to the level of *U6*. The forward primer sequences of the analyzed miRNAs are listed in Supplementary Table S1, and the reverse primers were supplied by the miRcute Plus miRNA qPCR Detection Kit.

### Immunostaining and histological methods

The cryosection immunostaining was performed as previously described^54^. The primary antibodies were anti-GFAP (DAKO, Z0334), anti-GFP (Abcam, ab13970), anti-PCNA (Sigma, P8825), anti-PSA-NCAM (Chemico, MAB5324), and anti-H2A.XS139ph (Genetex, GTX127340). The zebrafish p53 polyclonal antibody was generated by HuaAn Biotechnology (Hangzhou, China) as previously described. The secondary antibodies were anti-Rabbit IgG H&L Dylight 549 (EarthOx, E032320), anti-Rabbit IgG H&L Alexa Fluor 647 (Abcam, ab150143), anti-Chicken IgY H&L Alexa Fluor 488 (Abcam, ab150169), anti-mouse IgG H&L Alexa Fluor 488 (Abcam, ab150113), and anti-mouse IgG H&L Alexa Fluor 647 (Abcam, ab150115). Nuclei were stained by DAPI (BYT, C1002).

### TUNEL assay

The TUNEL assay was performed on freshly prepared cryosections of WT and *Δ113p53*^*M/M*^ zebrafish telencephalons at 16- and 24-months old using a fluorescein-based Roche In Situ Cell Death Detection Kit (Roche, 12156792910).

### Conditioned place avoidance (CPA) assay

The CPA assay described previously^46^ was modified according to our conditions. The experiment consisted of three periods: the Adaptation period, the Baseline period, and the Conditioning period. Starting from the Adaptation period, fish were transferred into individual tanks (180 × 90 × 75 mm) with a similar volume of fish water throughout the experiment. Each treatment and assay began at 9:00 every morning. In the Adaptation period (3 days), the tank was covered with white color. Each fish was separately put in the tank for 5 min/day to adapt the new environment. In the Baseline period (pre-electrical stimulation period) (6 days), the test tank was covered by two colors, one side with red color and the other with white color, and installed with four stainless steel flat electrodes along two long walls. During the Baseline data collection, fish activity was recorded for 5 min/day. In the Conditioning period (7 days), fish activity was recorded for 5 min/day before the electrical shock training. During Conditioning, a mild electrical shock (voltage: 1 V; electrical shock frequency: 0.8 s/1.0 s, electrical shock cycle: 1 min) was delivered each time the fish entered the white zone. The training was repeated again in 1-h interval. All of these records were analyzed by Any-Maze Video Tracking System. The time fish spent in each half of the tank (white or red color) in the Baseline period was compared to that in the Conditioning period. Each group contained eight fishes. The activity measurements in the CPA assay were analyzed by paired *t* test and two-way ANOVA measurement using the Graphpad Prism 5.

### Quantification and statistical analysis

Sample sizes were designed based on the routine genetic analysis in zebrafish studies. The investigators were blinded to group allocation during data collection and analysis. No data were excluded from the analyses. Male fish were used in the experiments for EDU-labeling, TUNEL assay, immunostaining (PCNA, γH2Ax), SA-β-gal staining, H_2_O_2_ detection, and CPA assay. Both male and female fish were used in the experiments for the expression of *Δ113p53* and cell lineage tracing. Fish were randomly sampled. The representative picture in each group was taken from at least three telencephalons. The experiments were repeated two to three times with similar results. The student’s two-tailed *t* test was applied in all statistical analysis, except the CPA experiments, in which paired *t* test was used within the group and two-way ANOVA analysis was used between the groups.

## Supplementary information

Figure S1

Figure S2

Figure S3

Figure S4

Figure S5

Figure S6

Supplementary figure legends and table S1
