# Supplementary figures and images for: Loss-of-function of p53 isoform Δ113p53 accelerates brain aging in zebrafish
Source: Cell Death Dis. 2021 Feb 4;12(2):151. doi: 10.1038/s41419-021-03438-9 (PMC7862496; doi:10.1038/s41419-021-03438-9)

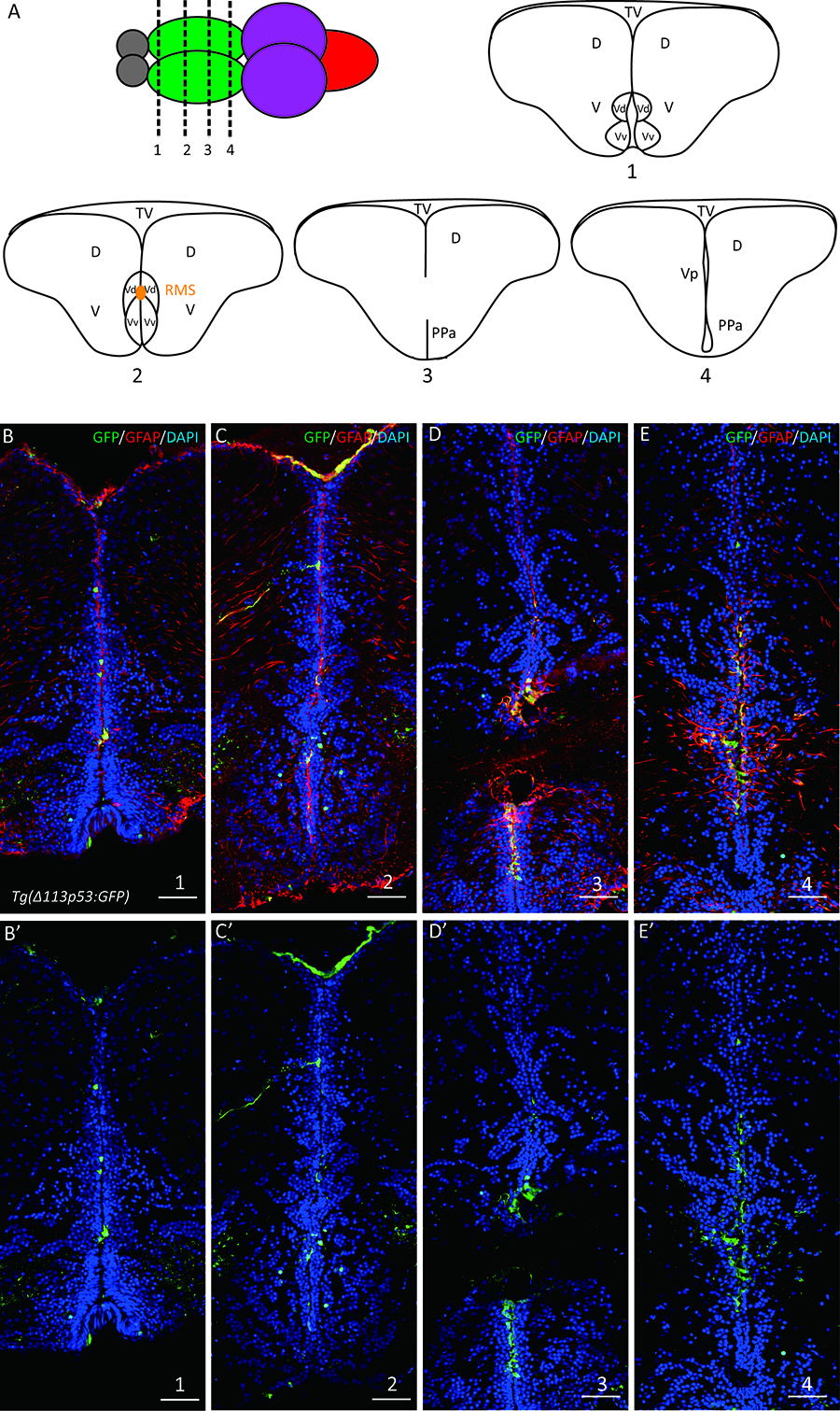

Supplement: Supplementary file 1 — Figure S1 [file 41419_2021_3438_MOESM1_ESM.tif]

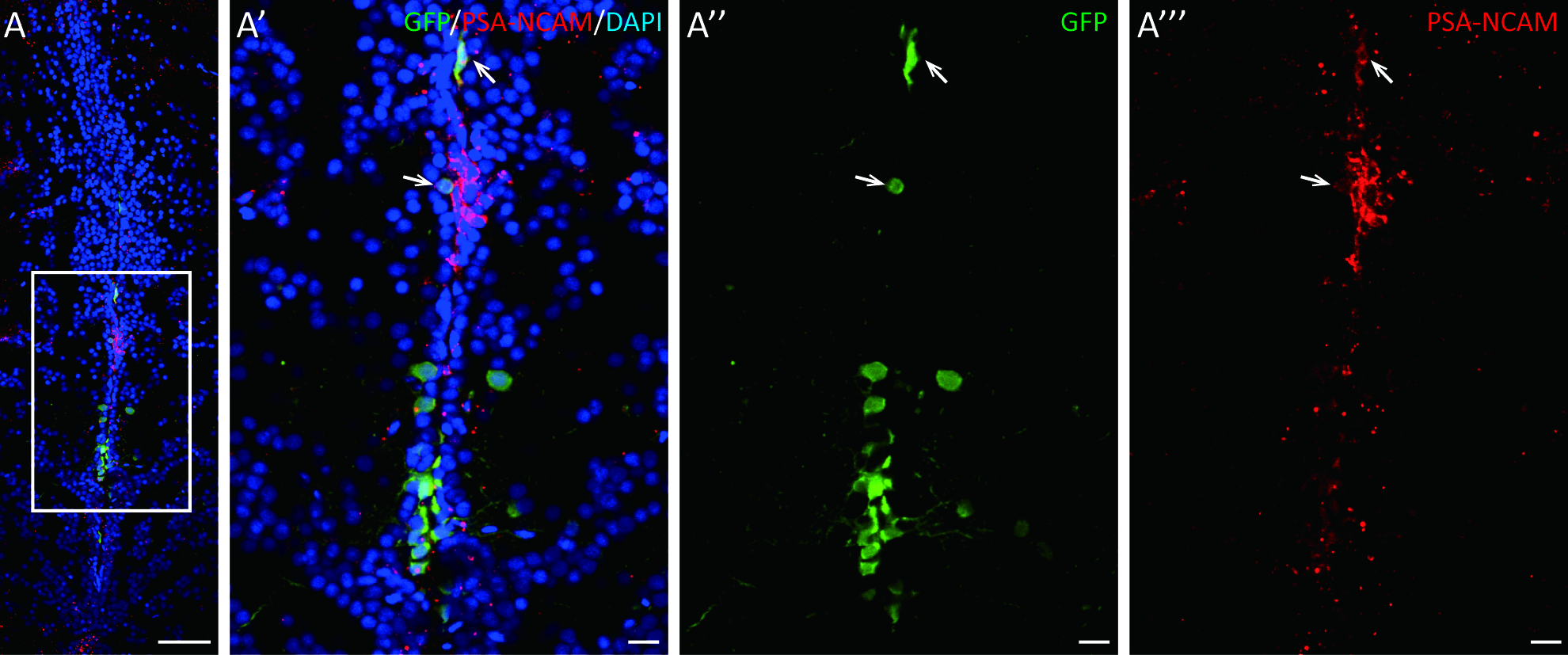

Supplement: Supplementary file 2 — Figure S2 [file 41419_2021_3438_MOESM2_ESM.tif]

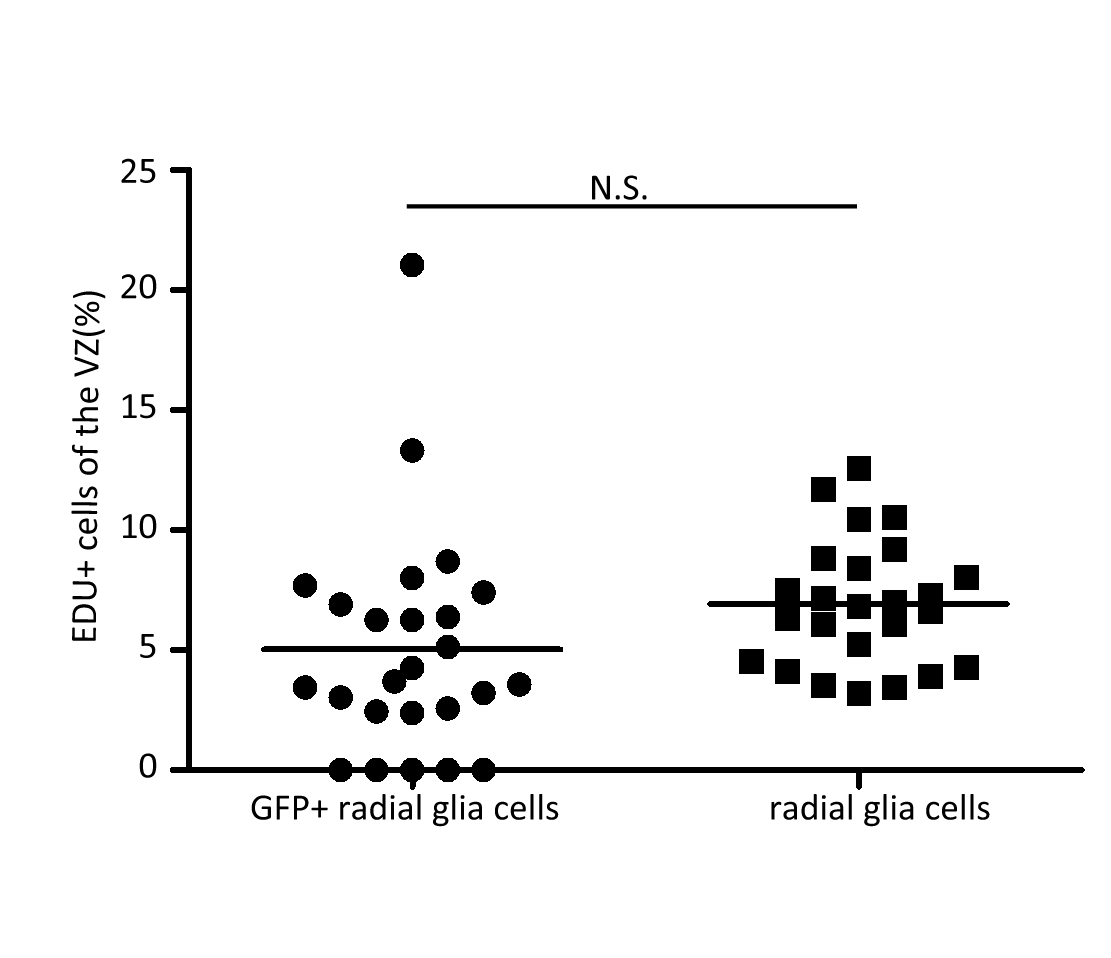

Supplement: Supplementary file 3 — Figure S3 [file 41419_2021_3438_MOESM3_ESM.tif]

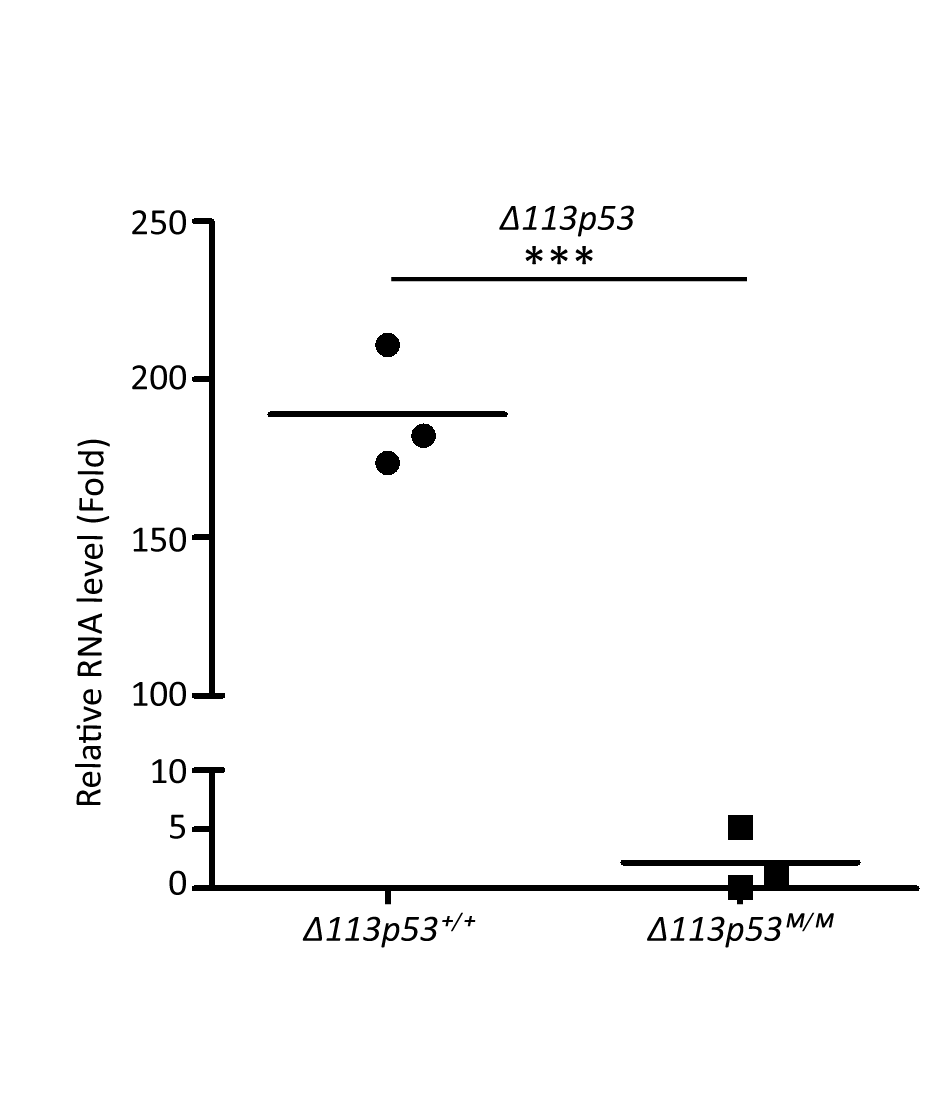

Supplement: Supplementary file 4 — Figure S4 [file 41419_2021_3438_MOESM4_ESM.tif]

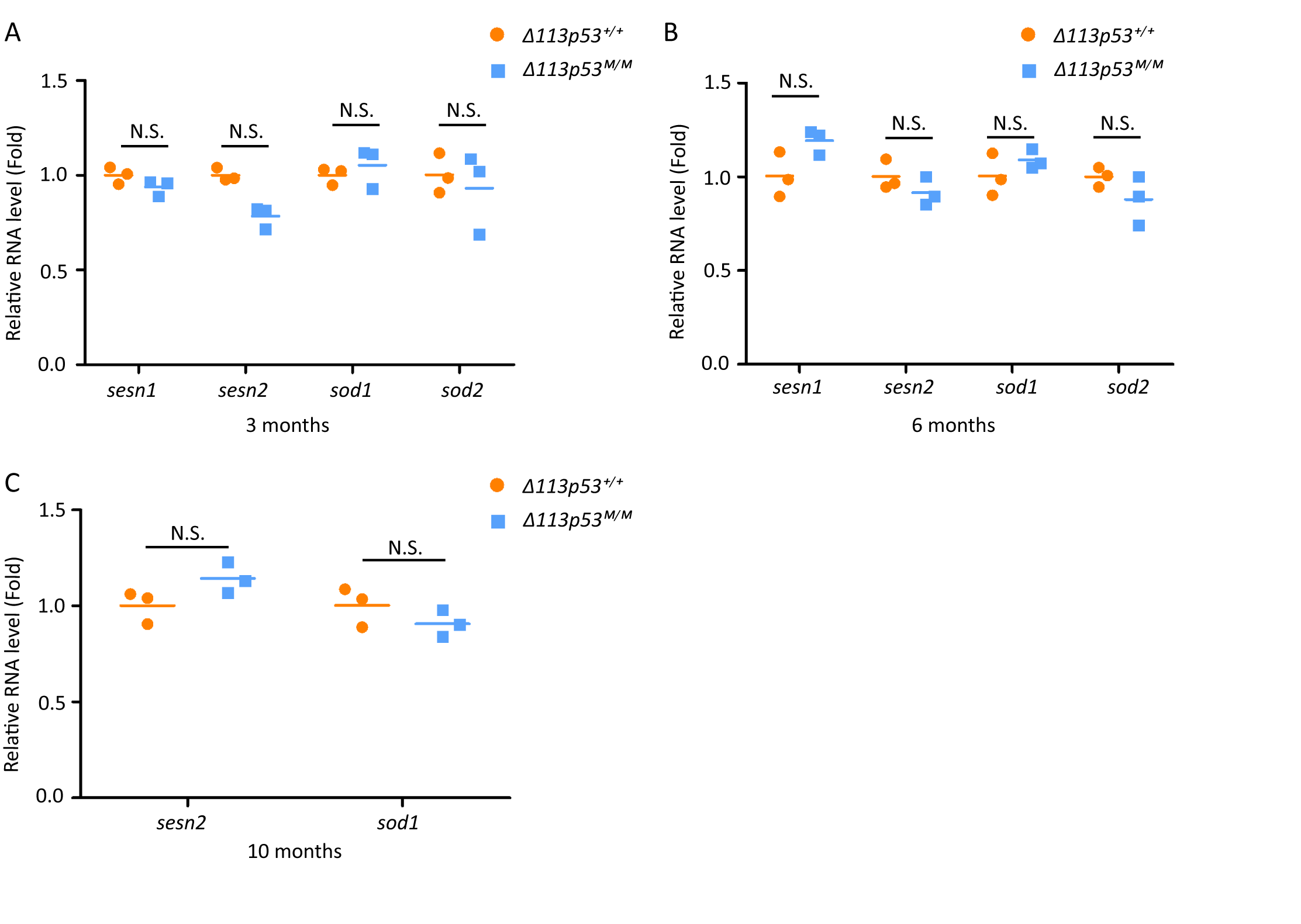

Supplement: Supplementary file 5 — Figure S5 [file 41419_2021_3438_MOESM5_ESM.tif]

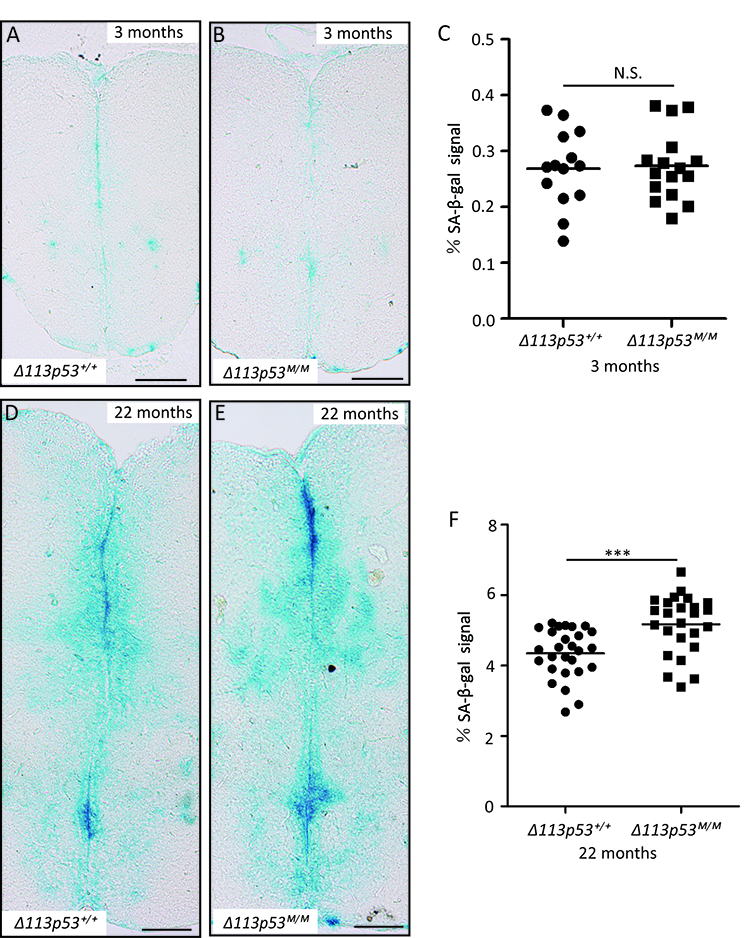

Supplement: Supplementary file 6 — Figure S6 [file 41419_2021_3438_MOESM6_ESM.tif]
