# Supplementary material for: Loss-of-function of p53 isoform Δ113p53 accelerates brain aging in zebrafish
Source: Cell Death Dis. 2021 Feb 4;12(2):151. doi: 10.1038/s41419-021-03438-9 (PMC7862496; doi:10.1038/s41419-021-03438-9)
Supplement: Supplementary file 7 — Supplementary figure legends and table S1 [file 41419_2021_3438_MOESM7_ESM.pdf]

Supplementary figure legends

Fig S1. *Δ113p53* expresses in the ventricular zone of zebrafish telencephalon. (A) Top left panel: A **s**chematic diagram representing zebrafish brain structure. The dotted lines with different number represent different positions in zebrafish telencephalon along the anterior to posterior axis. Gray color: olfactory bulb; Green color: telencephalon; Purple color: midbrain; Red color: hindbrain. Rest of panels: Diagrams representing cross sections of different regions in telencephalon corresponding to the positions as indicated by the dotted line in the top left panel. TV: telencephalic ventricle; D: dorsal telencephalic area; V: ventral telencephalic area; Vd: dorsal nucleus of V; Vv: ventral nucleus of V; RMS: rostral migratory stream; PPa: the anterior part of the parvocellular preoptic nucleus; Vp: the postcommissural nucleus of the ventral telencephalic area. **(B-E)** Cryosections of *Tg(Δ113p53:GFP)* telencephalons with different numbers corresponding to different regions as shown in **Figure S1A** were immunostained by anti-GFP (in green) and anti-GFAP (in red) antibodies. The nuclei were stained with DAPI (in blue). Scale bar, 50 μm.

Fig S2. Some of *Δ113p53* expresses in RMS-cells along the telencephalon ventricular zone. Cryosections of *Tg(Δ113p53:GFP)* telencephalon were immunostained by anti-GFP (in green) and anti-PSA-NCAM (in red). The nuclei were stained with DAPI (in blue). Framed area in **A** was magnified in **A'** (merged)**, A''** (GFP) and **A'''** (PSA-NCAM). White arrows: GFP^+/ PSA-NCAM $^+$ cells. Scale bar in **A**, 50 μm; Scale bar in **A', A'', A'''**, 10 μm.

Fig S3. Statistical analysis on the proportion of $EDU^+/GFP^+/GFAP^+$ cells among GFP

positive radial glia cells and the EDU$^+$/ GFAP$^+$ cells among total radial glia cells along the VZ in Figure 2A. EDU-labelled adult *Tg(Δ113p53:GFP)* telencephalons were immunostained by anti-GFP (in green) and anti-GFAP (in red) antibodies (Figure 2A). The proportion of EDU$^+$/GFP$^+$/GFAP$^+$ cells among GFP$^+$/GFAP$^+$ cells and the EDU$^+$/ GFAP$^+$ cells among total GFAP$^+$ cells along the VZ of *Tg(Δ113p53:GFP)* telencephalon were calculated from 6-7 sections/telencephalon in 4 telencephalons. Each dot represents the percentage of EDU$^+$/GFP$^+$/GFAP$^+$ cells among GFP$^+$/GFAP$^+$ cells or EDU$^+$/ GFAP$^+$ cells among GFAP$^+$ cells of ventricular zone in one section. The *P* values were represented by N.S. and asterisks. N.S., P >0.05.

Fig S4. Relative mRNA expression of *Δ113p53* in *Δ113p53*$^{+/+}$ and *Δ113p53*$^{M/M}$ mutant telencephalons. The total RNA was extracted from a pool of at least 6 telencephalons of *Δ113p53*$^{+/+}$ or *Δ113p53*$^{M/M}$ mutant. The average gene expression was normalized against *β-actin* and expressed as fold change.

Statistical analysis was performed on relevant data using Student's two-tailed t-test. The *P* values were represented by N.S. and asterisks. ***, $P< 0.001$.

Fig S5. The expression of some antioxidant genes in *Δ113p53*$^{+/+}$ and *Δ113p53*$^{M/M}$ zebrafish telencephalons at different ages. (A-C) Relative mRNA expression of *sesn1, sesn2, sod1, sod2* at 3 **(A)**, 6 (**B**) and 10 months-old (**C**) in the *Δ113p53*$^{+/+}$ or *Δ113p53*$^{M/M}$ telencephalons. The total RNA was extracted from a pool of at least 6 telencephalons in each group. The average gene expression was normalized against *β-actin*

and expressed as fold change.

Statistical analysis was performed on relevant data using Student's two-tailed t-test. N.S., $P>0.05$.

Fig S6. *Δ113p53*$^{M/M}$ zebrafish telencephalon has increased levels of cell senescence marker SA-β-gal with age. (A-F) Senescence-associated β-galactosidase (SA-β-gal) staining in the telencephalons of *Δ113p53*$^{+/+}$ (**A**, **D**) and *Δ113p53*$^{M/M}$ zebrafish (**B**, **E**) at 3 and 22 months-old as indicated. Scale bar, 100 μm. (**C**, **F)** The average SA-β-gal signal was quantified with Photoshop and presented as the percentage of pixels per unit area of the section measured in VZ. Each dot represents the average SA-β-gal signal in each section. About 3-5 sections were taken from the middle region of each telencephalon and at least 4 telencephalons were sampled in each group.

Statistical analysis was performed on relevant data using Student's two-tailed t-test. N.S., $P>0.05$; ***, $P<0.001$

Supplementary tables

Table. S1. The primer sequences and accession numbers of the analyzed genes in qRT-PCR.

Genes (Accession)	Primer sequences	
β-actin (NM_131031)	forward	CATTGGCAATGAGCGTTTC
	reverse	TACTCCTGCTTGCTGATCCAC
Δ113p53 (NM_001328588)	forward	ATATCCTGGCGAACATTTGGAGGG
	reverse	CCTCCTGGTCTTGTAATGTCAC
aldh4a1 (NM_201158)	forward	GAGCCCACAATAATCGAGAC
	reverse	CAGATTTGTCTTGGGGGAAG
gpx1a (NM_001007281)	forward	GCACCAGGAGAACTGCAAG
	reverse	TTCAGGAACGCAAACAGAGG
sesn1 (NM_001002660)	forward	GGGTGTGGACACTTCCATGC
	reverse	CCGGAACTGTCTCCAGAAGC
sesn2 (NM_001079975)	forward	ATGTTCAGCGCTCCGTCCTT
	reverse	CATCCAGAAGCTGCGCCATC
sod1 (NM_131294)	forward	TGGGTAATGTGACCGCTGAT
	reverse	ACTTTCCTCATTGCCACCCT
sod2 (NM_199976)	forward	ACTATGGTGCACTTGAGCCT
	reverse	CGCCATTGGGTGACAGATTT
p21 (NM_001128420)	forward	TCCCGCATGAAGTGGAGAAA
	reverse	GACGCTTCTTGGCTTGGTAG
sirt1 (XM_001334404)	forward	AAGGTGGACTGTGAAGCCAT
	reverse	ATCCTGCTTCATGGCTCTGT
e2f1 (XM_005174533)	forward	GATCTCCTGGCTCAGTCTCC
	reverse	CTTCTGCAGCTCCTGAAACC
cmyc (NM_131412)	forward	CACTCCACCTAACAGCTCCA
	reverse	GGTACCTCGACTCTGAAGCA
U6 （NM_001302466.1）	forward	TGCTCGCTACGGTGGCACA
	reverse	AAAACAGCAATATGGAGCGC
miR-34a (MI0001365)	forward	GCTGGCAGTGTCTTAGCTGGTTGT
miR-34b (MI0003690)	forward	GCTAGGCAGTGTTGTTAGCTGATTG
miR-34c (MI0004774)	forward	GCAGGCAGTGCAGTTAGTTGATTAC
